# Synthesis, Characterization and Optical Behavior of Nanocrystalline CoWO_4_

**DOI:** 10.3390/molecules30193843

**Published:** 2025-09-23

**Authors:** Reni Iordanova, Maria Gancheva, Iovka Koseva, Georgi Avdeev, Petar Ivanov

**Affiliations:** 1 Institute of General and Inorganic Chemistry, Bulgarian Academy of Sciences, Acad. G. Bonchev, bl. 11, 1113 Sofia, Bulgaria; reni@svr.igic.bas.bg (R.I.); ikosseva@svr.igic.bas.bg (I.K.); 2 Institute of Physical Chemistry, Bulgarian Academy of Sciences, “Acad. Rostislaw Kaischew”, Acad. G. Bonchev, str., bl. 11, 1113 Sofia, Bulgaria; g_avdeev@ipc.bas.bg; 3 Institute of Optical Materials and Technologies, “Acad. Jordan Malinowski”, Acad. G. Bonchev, str., bl. 109, 1113 Sofia, Bulgaria; petar@iomt.bas.bg

**Keywords:** ball milling, solid-state reaction, TEM, IR, UV–vis, blue–green emission, CIE coordinates

## Abstract

Nanocrystalline CoWO_4_ sampled were synthesized using a simple mechanochemical approach and a solid-state reaction, respectively. The formation of nanocrystalline CoWO_4_ was characterized by X-ray diffraction (XRD) and infrared spectroscopy (IR). The optical properties of the obtained samples were explored by diffuse reflectance UV–visible (DRS) and photoluminescence (PL) techniques. A milling speed of 850 rpm led to the direct synthesis of monoclinic CoWO_4_ with a short reaction time (1 h). The complete reaction did not occur in the solid-state synthesis. The obtained samples had monoclinic crystal systems with different lattice parameters. The average crystallite sizes of CoWO_4_ were in the range of 20 to 180 nm. The TEM investigation showed that the morphology of the CoWO_4_ particles differed depending on the preparation conditions. The values of the determined optical bandgap of CoWO_4_ were the range of 1.89 to 2.18 eV, according to diffusion reflectance spectroscopy in the ultraviolet-to-visible range. Broader blue–green emission spectra with peaks at 430 nm were observed for samples prepared via both routes. The CIE color coordinates of the CoWO_4_ samples lay in the blue and purple regions. The quantum yields of the CoWO_4_ samples synthesized after 1 h and 5 h milling times at 850 rom were 0.34 and 0.67%, respectively. This study proposes an affordable mechanochemical approach for blue–green phosphors that could possibly be used in various light-emitting diodes (LEDs).

## 1. Introduction

It is well known that nanostructured inorganic materials have excellent chemical and physical properties. Cobalt tungstate (CoWO_4_) is a compound belonging to the group of transition metal oxides with the general formula AWO_4_, where A = Co, Zn, Fe and Mn. It is crystallized at room temperature, with a wolframite-type structure, where Co and W atoms are surrounded by six oxygen atoms and form distorted octahedral units [1,2]. Both types of octahedral groups share two edges with neighboring polyhedral formations of the same type, forming alternating chains. CoWO_4_ is isomorphous to other transition metal tungstate formations such as NiWO_4_, FeWO_4_ and ZnWO_4_ [2,3]. CoWO_4_ has gained a lot of attention due to its high chemical and thermal stability and good catalytic [4,5,6,7,8], sensing [9], magnetic [10,11,12,13], electrode [6,14], electrical [12,15] and optical properties [10,11,12,13,14,16,17,18,19,20,21]. CoWO_4_ is a p-type semiconductor with a narrow optical bandgap between 2.3 and 2.95 eV, making it suitable for use in photoluminescent materials and photocatalysts [4,5,6,7,11,12,13,14]. In the literature, there are reports that the emission efficiency of CoWO_4_ is strongly dependent on the synthesizing process and defects in its structure, crystallite size and particle shape. Metal transition tungstate AWO_4_, with a wolframite-type structure, shows emissions in the blue or green range due to the metal charge transfer transition that occurs in WO_6_ [16,17,18,19,20,21,22,23]. The CoWO_4_ nanoparticles with a crystallite size of about 20 nm, as obtained by spray pyrolysis, exhibits a strong blue emission peak at 410–420 nm [17]. A broader emission profile with a peak maximum at 450 nm was reported for CoWO_4_ samples with crystallite sizes of 50 nm, as prepared via the hydrothermal route [11]. A sharp emission peak at the same wavelength was reported for a CoWO_4_ sample obtained by the molten salt method [18]. A CoWO_4_ sample with spherical particles exhibited broad intrinsic PL emissions between 400 and 600 nm with major peaks around 468 nm (blue–green) and 530 nm (green) [12,19,20,21,22,23]. According to P. Taneja et al., the emission peak at 470 nm was associated with the reduction in the phase formation of [WO_6_] units in the CoWO_4_ nanostructure, while the shoulder at 495 nm was related to the formation of defects by oxygen vacancies [7]. CdWO_4_, with besom-like particles, shows blue–green light emissions centered at 475 nm [23]. The strong green peak around 495 nm, with a shoulder at 530 nm, is typical of CoWO_4_ samples that were formed with a nanowire morphology [20]. Such investigations are being conducted to increase the effectiveness of these materials in the abovementioned areas. Such improvements can be achieved through changes in the method of synthesis (new method) that is used or through a modification in the crystal structure. Various chemical and physical techniques have been used to prepare CoWO_4_ through a solid-state reaction [15], hydrothermal approaches [4,7,11,16], solution methods [1,5], co-precipitation methods [6,8,12,14,21] and molten salt synthesis approaches [18]. Only Xiao et al. has used the mechanical activation of a mixture of Co_3_O_4_ and WO_3_ in absolute ethanol for 6 h, followed by heat treatment at 900 °C, to obtain CoWO_4_ [15]. Up to the present day, there have not been investigations into the use of ball milling in dry atmospheres for the preparation of this phase. Mechanochemical synthesis in chemical processes is popular, allowing different functional nanoparticle materials to be synthesized; these materials control phase composition, morphology and the degree of crystallization. This approach is favored because it eliminates the need for organic additives and enables the acquisition of large amounts of material under ambient conditions; moreover, chemical reactions often occur faster than traditional solid-state reactions and wet solution methods. Furthermore, milling conditions (speed, time, mass ratio, atmosphere, milling materials, etc.) create new defects and surfaces in the treated solids, improving their properties. We reported on the optical properties and particle shapes of the inorganic mixture oxides of AMO_4_ (A = Ba, Sr, Zn and M = Mo and W), as synthesized by direct mechanochemical treatment [24,25,26,27,28,29]. We demonstrated that the particle morphology of SrWO_4_, CaWO_4_ and BaWO_4_ samples were characterized by spherical, hexagonal and quasi-hexagonal forms, depending on the ball milling conditions used (i.e., milling time and speed) [25,26,29]. We established that higher milling speeds led to fast synthesis and to the formation of different types of defects in the crystal phases [28,29]. We reported that a milling speed of 850 rpm led to the formation of deformation structural groups and to the occurrence of oxygen vacation in BaWO4 [29]. BaWO_4_ samples with microwave dielectric properties were prepared by combining mechanochemical treatments with additional thermal treatments [30]. On other hand, this approach was applied to obtain a sample of BaWO_4_ that was doped with Eu_2_O_3_ to ensure that it had good photoluminescence [31]. S. Balamurugan et al. demonstrated that dry ball milling resulted in the direct synthesis of pure CaWO_4_, while wet ball milling using ethanol and/or water as a solvent did not yield pure CaWO_4_ at room temperature [32]. Our previous results showed that the single phase of MgWO_4_ was produced through 850 °C thermal treatment of a mechanically activated mixture of MgCO_3_. 3H_2_O and WO_3_ [33]. The mechanochemical treatment was used to obtain materials with tunable photoluminescent properties [34,35,36]. In this study, we investigated the optical properties of CoWO_4_, as obtained by both methods of preparation: mechanochemical synthesis and solid-state reaction. The structural and optical properties are compared.

## 2. Results and Discussion

### 2.1. Phase Formation and Characterization

#### 2.1.1. X-Ray Diffraction Analysis

**Figure 1:**
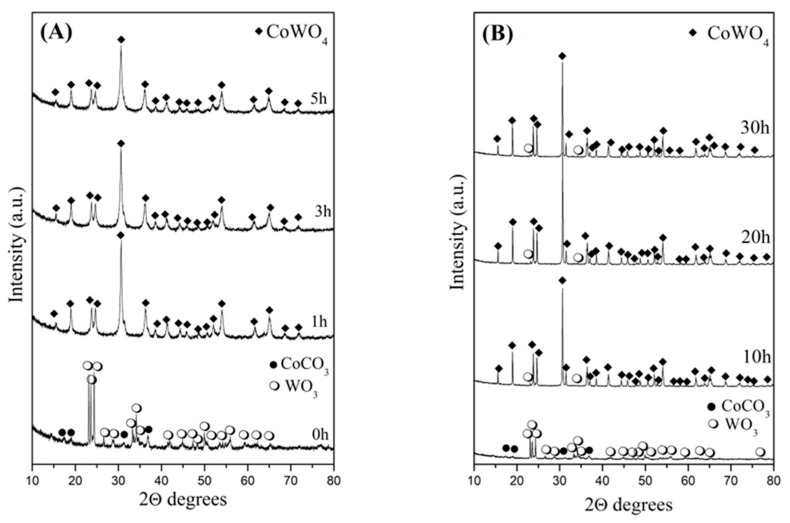
(**A**,**B**). X-ray diffraction patterns of CoWO_4_ phase formation prepared by mechanochemical synthesis and a solid-state reaction.

The reaction time and phase formation of CoWO_4_, created using a mechanochemical treatment and a solid-state reaction, were monitored using X-ray diffraction analysis. Figure 1A,B show the XRD patterns of the samples subjected to ball milling and 700 °C thermal treatment, respectively. The X-ray diffractogram of the initial mixture exhibits the main lines of both components: hexagonal CoCO_3_ (PDF-98-005-2377) and monoclinic WO_3_ (PDF-98-001-6080). These data can be obtained free of charge via https://www.icdd.com/. The mechanochemical treatment for 1 h led to appearance of new diffraction lines that are typical of monoclinic CoWO_4_ in the P12/c1 group (PDF-98-001-5851) (Figure 1A). In Figure 1A, it can be seen that secondary phases or impurities are not distinguishable after mechanical activation through milling times of 3 and 5 h. This is indicative of the high phase purity of ball-milling-synthesized CoWO_4_. This result is in good agreement with the notion that ball milling is an effective approach for rapidly synthesizing AWO_4_-type compounds at room temperature [24,25]. The X-ray diffraction data of the sample heated at 700 °C for different durations are shown in Figure 1B. Strong diffraction lines, characteristic of monoclinic CoWO_4_ (PDF-98-001-5851), were observed after annealing at 700 °C for 10 h. But the low-intensity profiles of unreacted WO_3_ were also detected at 23.15 and 34.50°. In the figure, one can see that the intensity of the CoWO_4_ diffraction peaks increases with annealing time. An unreacted WO_3_ sample was still observed after a prolonged calcination time of up to 30 h, and a single phase of CoWO_4_ did not form. 

This study shows that the interaction between CoCO_3_ and WO_3_ is more effectively facilitated by mechanical activation with higher milling energy in comparison to that with lower milling energy. It is notable that the diffraction peaks are broader, with lower intensity for the mechanochemically synthesized CoWO_4_ sample than for the sample prepared through a solid-state reaction. The phase composition, lattice parameters and average crystallite size of the synthesized samples were evaluated using High Score Plus option, version 4.0; the results are presented in Table 1. The extended milling duration resulted in an increase in the values of unit cell volume (V) and the lattice parameters (b and c). We also observe that the strain in CoWO_4_ decreases with increasing milling time; this could be explained by the higher crystallite size. The sample produced using the solid-state route possessed lower cell volume (V) and microstrain and higher crystallite size. The strain value was found to be significantly influenced by increases in the crystallite size. 

#### 2.1.2. TEM Analysis

**Figure 2:**
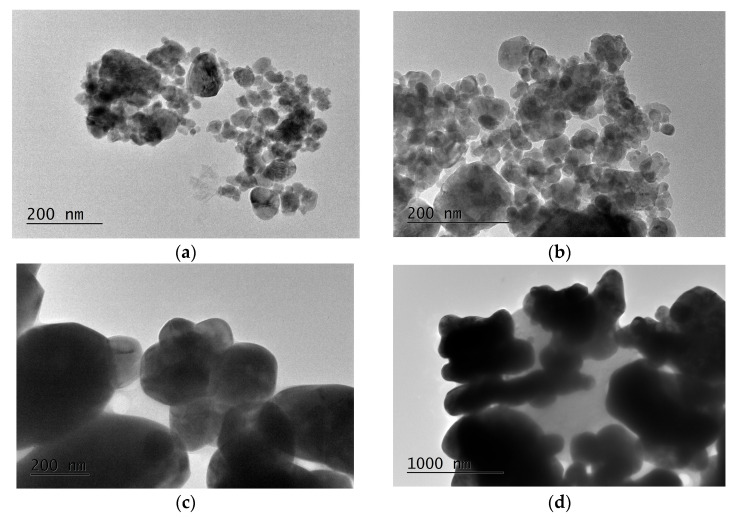
TEM micrographs of CoWO_4_ obtained by mechanochemical synthesis (**a**,**b**) and solid-state reaction (**c**,**d**).

Both the mechanical activation and thermal treatment greatly impacted the particle morphologies of the final products. Figure 2 displays TEM images of the CoWO_4_ samples that were synthesized with a 5 h milling time at a milling speed of 850 rpm (a and b) and through the solid-state reaction (c and d). The TEM images, at low (a; ×25,000) and high (b; ×40,000) magnifications, show the spherical particle morphologies, with slight agglomerations; this morphology is typical of samples obtained using the ball milling process. The TEM images presented in Figure 2a,b illustrate that the particle sizes range from 10 to 30 nm. This particle form is similar to that established in other materials prepared by mechanochemical treatments [25,27]. 

Figure 2c,d depict the particle morphologies of CoWO_4_ samples obtained by a solid-state reaction at low (c; ×25,000) and high magnifications (d; ×40,000). The edges of the particles are noticeable regardless of the agglomeration, which indicates that CoWO_4_ consists of hexagonal, oval, spherical and irregular grains. The large agglomeration in the sample is due to the longer reaction time (700 °C, 30 h). The sizes of the particles are above 100 nm. There is a visible difference in the shapes of the particles from both samples. It should be noted that the surfaces of both the CoWO_4_ samples are very smooth.

#### 2.1.3. Infrared Investigation

**Figure 3:**
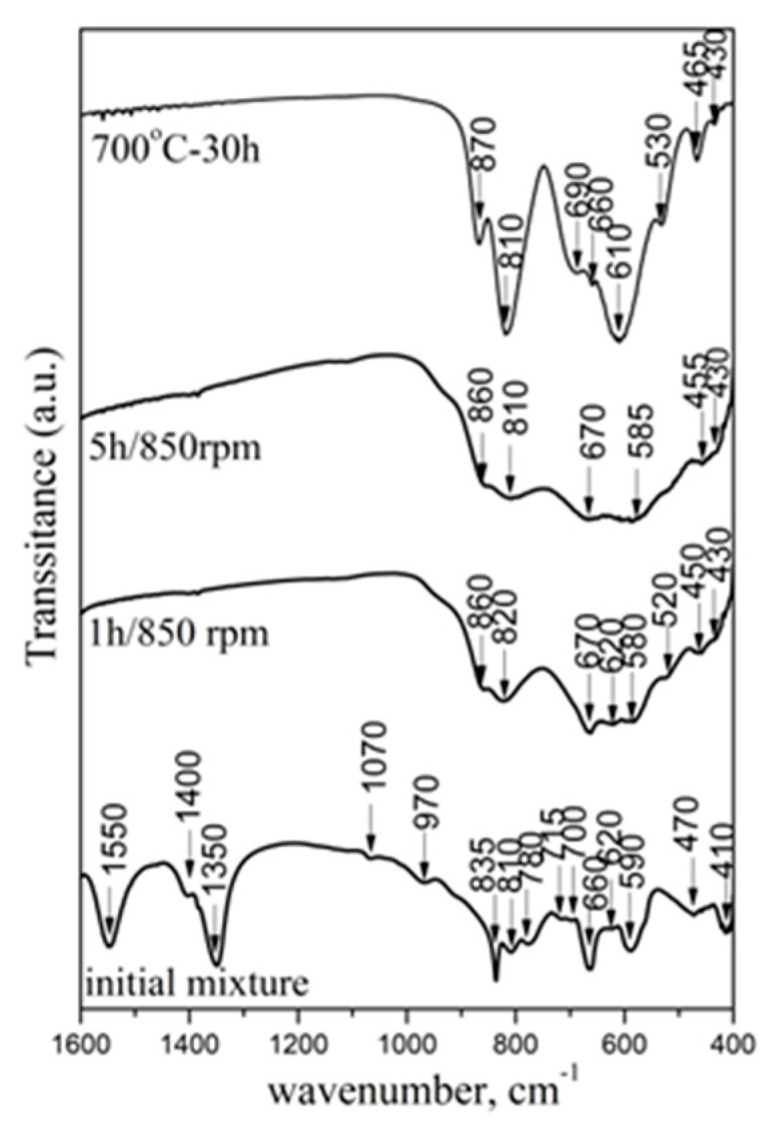
IR spectra of initial mixture: CoWO_4_ obtained by mechanochemical synthesis and solid-state reaction.

The phase formation of CoWO_4_ was confirmed using IR spectroscopy (Figure 3). The IR spectrum of the initial mixture exhibits absorption bands typical of both reagents. The bands at 970, 810 and 780 cm^−1^ were due to the vibration of the W-O-W bridges of WO_6_, used in building the crystal structure of WO_3_ [37]. The absorption bands above 1000 cm^−1^ (bands at 1550, 1400, 1350 and 1070 cm^−1^) are attributed to the ν_3_ and ν_1_ stretching vibrations of the (CO_3_)^2−^ groups. The vibration of the ν_2_ (835cm^−1^) and ν_4_ bending modes of the same units (at 715, 620, 660 and 590 cm^−1^) were also registered [38,39].

After 1 h, the mechanical treatment to the disappearance of the bands of the initial precursors. A set of new absorption bands at 860, 820, 660 (670), 520, 450 and 430 cm^−1^ were registered. This is an indication of creative new structural units due to the interaction between CoCO_3_ and WO_3_. The bands at 860 and 820 cm^−1^ were due to the vibration of the WO_2_ entity present in the W_2_O_8_ groups, which exists in the structure of CoWO_4_. The bands at 670 and 585 cm^−1^ are typical of a two-oxygen bridge (W_2_O_2_) and are present due to the asymmetric stretching of the same units. In this case, the vibration of Co-O in the CoO_6_ polyhedral form falls in the absorption range below 500 cm^−1^ [6,14,21,24,26]. The lower intensity of the IR bands of the mechanochemically synthesized CoWO_4_ sample is related to the lower crystallite size and is in a good agreement with the results of the X-ray diffraction analysis. The increasing milling time up to 5 h led to disappearance of the shoulders at 620 and 520 cm^−1^. The IR spectrum of the sample prepared by solid-state reaction exhibits stronger intensity bands due to the higher crystallite size. It was noted that the positions of the bands at 670 and 585 cm^−1^ changed to higher wavenumbers: 690 and 610 cm^−1^. The additional band at 660 cm^−1^ was present due to the vibration in the Co-O bonds in the crystallite phase [13,34]. The positions and intensities of the IR bands in CoWO_4_ were found to be in correlation with those reported by Y.L. Oliveira et al. [14].

### 2.2. Optical Properties of CoWO_4_ Obtained Through Mechanochemical and Solid-State Approaches

#### 2.2.1. UV–Vis Defuses Reflectance

**Figure 4:**
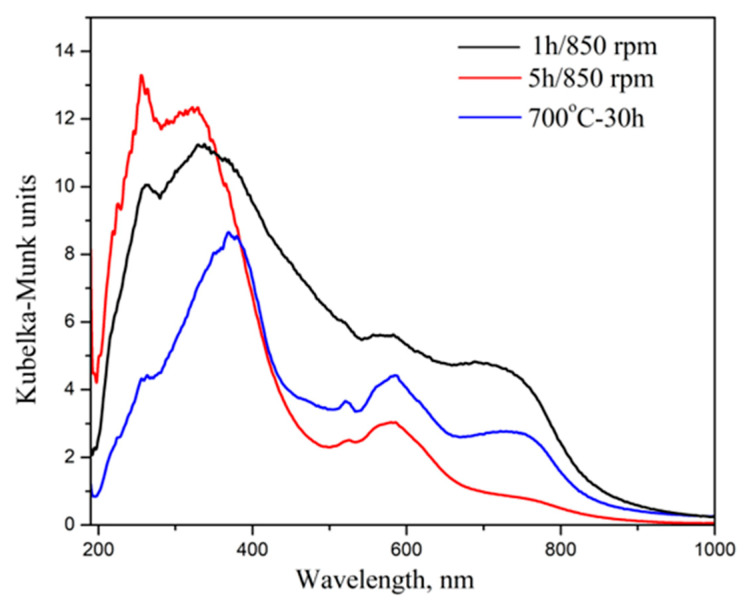
The Kubelka–Munk function of the CoWO_4_ obtained by mechanochemical synthesis and solid-state reaction.

The study of optical properties is important in informing LED applications; UV–vis absorption is directly related to the energy bandgap of the crystal phase. The optical characteristics of the CoWO_4_ samples obtained through mechanochemical treatment and the solid-state reaction were studied by measuring diffuse reflectance and through photoluminescence spectroscopy. The diffuse reflectance spectra were transformed into a Kubelka–Munk function; the results are shown in Figure 4. The Kubelka–Munk spectra of all the samples exhibit an absorption band in the UV range from 250 to 370 nm and in the visible range from 515 to 760 nm. The peaks in the first range are a result of the charge transfer from oxygen to tungstate atoms in WO_6_ units [12,14,21]. It was noted that the intensity of the peak at 255 nm is higher than that for the CoWO_4_ sample that was obtained after 5 h milling time at 850 rpm. The shape and position at the second peak, 330 nm, were changed for the other samples. The band position shifted up to 370 nm for the sample synthesized at using the solid-state reaction (700 °C for 30 h); for this sample, the peaks at the higher wavelengths (515, 580 and 740 nm) have good resolutions and have higher compound intensities. The first peak at 515 nm is due to the following electron transfer: Co (3d) → W (5d) [12]. The absorption peaks at 580 nm and 740 nm are typical of forbidden d–d transitions in Co^2+^ ions in CoO_6_ octahedral units [11,12,40]. 

**Figure 5:**
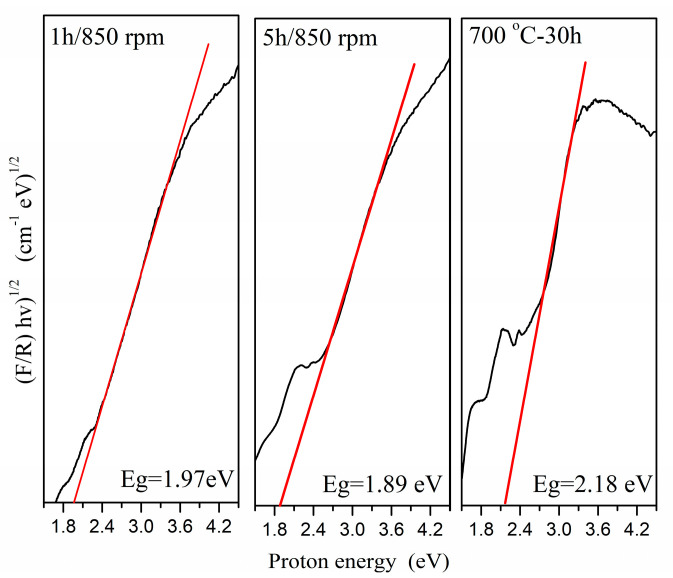
The Tous plots of (F/R)hν)^1/2^ versus the photon energy (eV) of the CoWO_4_ obtained by mechanochemical synthesis and solid-state reaction.

The optical bandgap (Eg) is one important parameter that can be used to forecast the suitability of these materials for optoelectronic applications in semiconductor materials. The optical bandgap can be estimated based on Tauc’s equation; the results are presented in Figure 5. From the figure, it is clear that the CoWO_4_ powders prepared by ball milling have a relatively lower bandgap value than the CoWO_4_ samples obtained using a solid-state reaction and the other methods reported in the literature [4,5,7]. Annealing reduces defects in structures, amplifying the nature of the crystals and increasing the crystallite size (Table 1). This is probably the explanation for the higher value of the optical bandgap. The reduced optical bandgaps of the samples obtained through mechanochemical methods can be attributed to their smaller crystallite size and to the formation of defects in their structures. The short reaction time of the CoWO_4_ formation (1 and 5 h), with a milling speed of 850 rpm, led to the production of structural defects (Table 1); as a result, the electron concentration increases. This leads to the presence of some localized electronic states near the conduction band. Therefore, mechanochemical treatment is directed to the crystal phase with a lower optical bandgap. In this case, we establish that the crystallite size and defects can be considered as the major factors in the value of the optical bandgap. 

#### 2.2.2. Photoluminescence Emission Spectra

**Figure 6:**
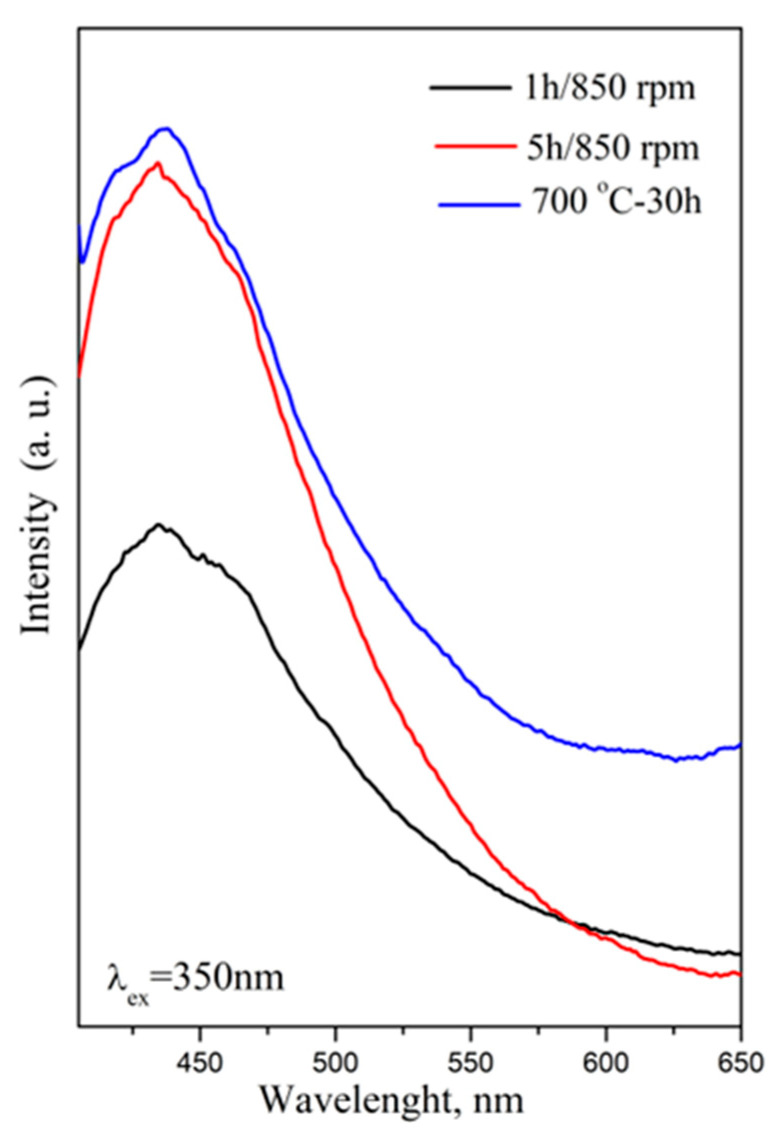
Photoluminescent emission spectra of the CoWO_4_ samples obtained by mechanochemical synthesis and solid-state reaction.

To obtain a more comprehensive analysis, a PL measurement was carried out at room temperature. The emission spectra of the CoWO_4_ powder samples obtained using the mechanochemical and solid-state approaches, recorded under an excitation wavelength of 350 nm, are illustrated in Figure 6.

The dominant emission band is located at 435 nm; the additional two weak shoulders at 416 and 463 nm were also visible. The position of the emission peak is closer to those reported in the literature, as related to the charge transfer transitions of the WO_6_ group [19,20,21,22,23]. The sample obtained using the solid-state reaction exhibits stronger emissions than the sample prepared with a 1 h milling time at 850 rpm. This fact can be attributed to the higher crystallite size and the lower level of crystal defects (Table 1). The result is in good agreement with previous investigations: the intensity of the PL emission depends on the crystallinity of the materials [23,25,27]. It was noted that the sample prepared using a short milling time (1 h milling time at milling time of 850 rpm) possesses the lowest intensity; this is probably due to the presence of more defects in the crystal structures, as well as the lower crystallite size. We can conclude that the crystallite size and the method of preparation influence the PL properties of CoWO_4_. 

**Figure 7:**
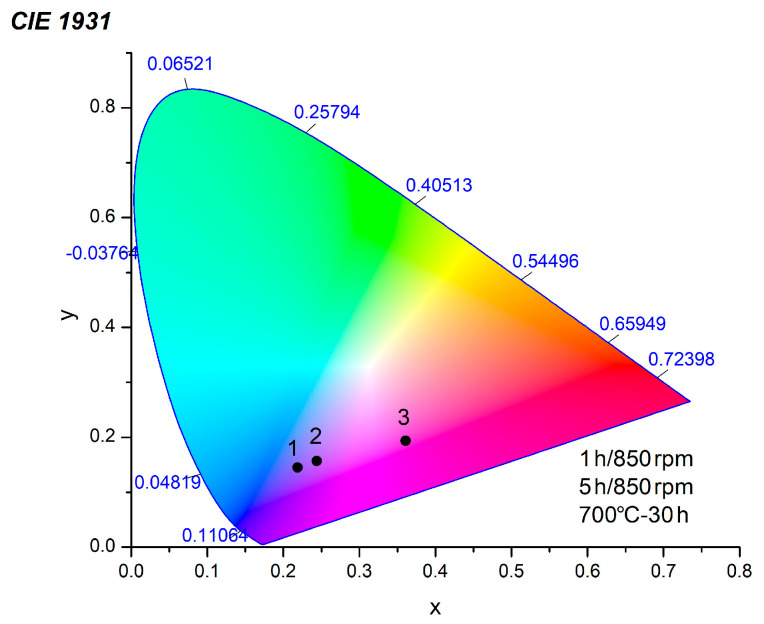
CIE diagram of the CoWO_4_ samples obtained by mechanochemical synthesis and solid-state reaction. 1 is sample obtained after 1 h milling time using milling speed of 850 rpm, 2 is sample obatined after 5h milling time using milling speed of 500 rpm and 3 is sample prepared by solid state reaction.

The CIE chromaticity coordinates (x, y) serve as a significant factor for the color emission of the obtained materials. They were calculated from the emission spectra and are highlighted in the CIE diagram in Figure 7. The CIE chromaticity coordinates of the mechanochemically obtained samples lie in the blue region under an excitation wavelength of 350 nm. A slight shift to the light region was observed; this was seen to depend on the duration of the milling treatment (Table 2). The CIE coordinates of the CoWO_4_ prepared using the solid-state reaction fall into the purple-highlighted area. 

The quantum yield (QY) (%) is related to the number of photons absorbed (a) and the number of photons emitted by the sample. As can be seen from Table 2, certain values (quantum yield (QY), luminescent lifetimes (τ_1,2_ and _eff_ [ns]) and the effective luminescent half-life $T_{1/2}^{eff}$) were higher for the CoWO_4_ samples obtained after 5 h at a milling speed of 850 rpm using a solid-state reaction in comparison with the samples produced with 1 h of milling. Therefore, the higher value of the quantum yield and the other PL parameters is probably associated with a more pronounced structural nature, as resulting from the longer reaction time.

## 3. Materials and Methods

### 3.1. Direct Mechanochemical Synthesis

Cobalt carbonate (CoCO_3_) (Merck KGaA, Amsterdam, The Netherlands, 99.9% purity) and tungstate oxide (WO_3_) (Merck KGaA, Amsterdam, The Netherlands, 99.9% purity) are used as raw materials. The initial mixture was in a stoichiometric ratio, 1:1, which corresponds to the crystal CoWO_4_ phase which was activated in the planetary ball mill (Fritsch premium line, Pulverisette No 7, FRITSCH GmbH, Idar-Oberstein, Germany). The ball milling treatment was carried out at milling speeds of 850 rpm at room temperature; the ball-to-powder weight ratio was 10:1. As guided by our earlier research, the process was performed in 15 min increments with 5 min rest intervals to reduce the temperature during the milling process [24,25,26,27,28,29]. 

### 3.2. Solid-State Synthesis

The initial mixture of the same reagents (CoCO_3_ and WO_3_) in stoichiometric ratio of 1:1 was homogenized in agate mortar for 10 min. Subsequently, the mixture was transferred to an aluminum crucible (Tehem, Sofia, Bulgaria) and heated at 700 °C for different durations (10, 20 and 30 h) in an electrical furnace Nebertherm LH117PN2 (Nabertherm GmbH, Lilienthal, Germany). Finally, the synthesized samples were naturally cooled to room temperature in the furnace.

### 3.3. Characterization

The XRD powder patterns were observed using Bruker D8 Advance X-ray powder diffractometer (Bruker, Karlsruhe, Germany), equipped with a CuKa radiation source (1.542 Å) and Lynx Eye PSD detector. The crystalline phase was identified using the HighScore Plus option version 4.0 (Almelo, NL Almelo, Netherlands). The morphologies of the powders were observed using a transmission electron microscope, JEM 2100 (JEOL, Tokyo, Japan), with a GATAN Orius 832 SC1000 CCD camera (AMETEK, Berwin, PA, USA) at an accelerating voltage of 200 kV. In order to prepare the specimen for TEM analysis, the sample was ground in an agate mortar and then ultrasonically treated for six minutes until it reached an ethanol suspension. A droplet of the suspension was coated on a standard carbon film on a Cu grid. Infrared spectra were registered in the range 1200–400 cm^−1^ on a Nicolet-320 FTIR spectrometer (Malvern GB, Madison, WI, USA) using the KBr pellet technique with spectral resolution of 2 nm. The diffuse–reflectance spectra were recorded with a Thermo Evolution 300 UV–vis Spectrophotometer (Malvern GB, Madison, WI, USA) equipped with a Praying Mantis device. For capturing the background data, a Spectralon (Malvern GB Madison, WI, USA) was used. The optical absorption band was calculated based on Tauc’s equation, *αhν = A(hν − Eg)^n^*; here, α is the absorption coefficient, A is the absorption constant, h is Plank’s constant, and ν is the photon frequency [41]. In the mentioned relation, n represents the type of semiconductor charge transition. The value of n is related to the characteristics of the electronic transition type in the semiconductors and *n* = 0.5 for a direct allowed transition; *n* = 2 for an indirect allowed transition; *n* = 3 for an indirect forbidden transition; and *n* = 3/2 for a direct forbidden transition. CoWO_4_ is known as a direct transition metal oxide; therefore, the value of *n* is 0.5. The PL emission spectra were measured on a Horiba Fluorolog 3–22 TCS spectrophotometer (Horiba Jobin Yvon S.A.S, Longjumeau, France), equipped with a 450 W Xenon Lamp as the excitation source. The quantum yield, luminescent lifetime and effective luminescent half-life were determined with the aid of a Quanta-Phi F-3029 integrating sphere (Horiba Jobin Yvon S.A.S, Longjumeau, France). The fluorescence lifetimes were measured through time-correlated single-photon counting (TCSPC) using a NanoLED pulsed excitation source (Horiba Jobin Yvon S.A.S, Longjumeau, France). 

## 4. Conclusions

This work examined the impact of the reaction time on the photoluminescence performance of CoWO_4_ samples produced through heat treatments at 700 °C, with mechanochemical synthesis. We demonstrated that mechanical activation significantly reduces the phase formation time for CoWO_4_. XRD and IR analyses were used to verify the formation of CoWO_4_ with a wolframite-type structure. The results of the TEM investigation demonstrated that spherical particles were observed in the CoWO_4_ sampled created with mechanochemical activation. The particles had different shapes in the CoWO_4_ sample obtained through a solid-state reaction.

As the reaction time increased, the optical bandgap of the CoWO_4_ samples increased from 1.89 to 2.18 eV. The different methods of preparation (1 and 5 h milling time; milling speed of 850 rpm) and reaction times (700 °C for 30 h) modified the lattice parameters, crystallite size and microstrain of CoWO_4_, affecting the emission intensities. The CoWO_4_ obtained using a solid-state reaction exhibited a stronger blue–green emission, with CIE coordinates that fall in the purple range. The blue region of the CIE diagram, with different coordinate positions, was observed for the mechanochemically prepared CoWO_4_ samples. These results suggest that the as-obtained CoWO_4_ samples could be a good choice for LED lighting applications.

## Figures and Tables

**Table 1:** Structural parameters, strain and crystallite size of CoWO_4_ prepared by mechanochemical synthesis and solid-state reaction. Parentheses represent the error in the calculation of the lattice parameters.

Samples	V/10^6^ pm^3^	a/Å	b/Å	c/Å	Strain %	Crystallite Size, nm
CoWO_4_-1 h/850 rpm	131.59	4.6630 (±6)	5.6899 (±7)	4.9599 (±6)	0.107	17
CoWO_4_-5 h/850 rpm	132.64	4.6593 (±2)	5.7141 (±1)	4.9810 (±1)	0.069	22
CoWO_4_-700 °C-30 h	131.19	4.6679 (±1)	5.6808 (±1)	4.9473 (±1)	0.008	178
(2 wt% WO_3_)	-	-	-	-	-	-
CoWO_4_ (PDF-98-0015851)	131.52	4.6700	5.6870	4.9520	-	-

**Table 2:** CIE color coordinates (x and y), quantum yield (QY), luminescent lifetime (τ [ns]) and effective luminescent half-life $T_{1/2}^{eff}$ [ns] of the CoWO_4_ samples obtained by mechanochemical synthesis and solid-state reaction.

Samples	x	y	QY %	τ [ns]	$T_{1/2}^{eff}$ [ns]
CoWO_4_, 1 h, 850 rpm	0.2186	0.1447	0.34	$\tau_{1}$ = 0.43, $\tau_{2}$ = 1.60, $\tau_{eff}$ = 0.72	0.40
CoWO_4_, 5 h, 850 rpm	0.2439	0.1569	0.67	$\tau_{1}$ = 0.83, $\tau_{2}$ = 3.00, $\tau_{eff}$ = 0.94	0.65
CoWO_4_, 700 °C, 30 h	0.3609	0.1938	0.60	$\tau_{1}$ = 0.75, $\tau_{2}$ = 2.56, $\tau_{eff}$ = 0.86	0.60

## Data Availability

The original contributions presented in this study are included in the article.
